# Comparison of semen preservation methods and *in vitro* fertilisation embryo quality in Simmental cattle

**DOI:** 10.5713/ab.250410

**Published:** 2025-11-25

**Authors:** Kai Hu, Fei Huang, Hong Chen, Hui-Min Qu, Xiao-Peng Li, Xue-Yan Wang, Jie-Ru Wang, Peng Niu, Di Fang, Qing-Hua Gao

**Affiliations:** 1 College of Animal Science and Technology, Tarim University, Alar, China; 2 College of Life Science and Technology, Tarim University, Alar, China; 3 Key Laboratory of Tarim Animal Husbandry Science and Technology, Xinjiang Production & Construction Corps, Alar, China

**Keywords:** 0°C Refrigeration, Embryo Quality, *In Vitro* Fertilisation, Liquid Nitrogen-frozen, Semen, Sperm Quality

## Abstract

**Objective:**

Semen quality plays a crucial role in embryo formation in cattle. This study aims to compare the effects of 0°C refrigerated semen and liquid nitrogen-frozen semen on the quality of *in vitro* fertilized embryos in Simmental cattle.

**Methods:**

Semen from six bulls was devidedt equally into two groups. One was diluted with cold storage solution and stored at 0°C for 72 h; the other with cryopreservation solution and preserved in liquid nitrogen for 72 h. Post-storage, sperm quality (motility, progressive motility via computer-assisted sperm analysis, and acrosome/plasma membrane integrity) was assessed, along with malondialdehyde, reactive oxygen species, adenosine triphosphate, and mitochondrial membrane potential levels. For *in vitro* fertilisation (IVF), oocytes from Simmental cows (>50 per group) were inseminated with the two semen groups. At 45 h post-IVF, embryo cleavage rate was recorded; on day 8, blastocyst rate was determined. Embryos then underwent BrdU staining (proliferation) and TUNEL staining (apoptosis), with proliferation and apoptosis levels quantified via marker genes.

**Results:**

The results showed that compared with liquid nitrogen-frozen semen, 0°C refrigerated semen exhibited significantly higher sperm motility, forward motility rate, acrosome integrity, and plasma membrane integrity (p<0.05). *In vitro* fertilized embryos also exhibited significant increases in quality and quantity (cleavage rate and blastocyst rate) (p<0.05), and embryos derived from 0°C refrigerated semen contained significantly more proliferating cells (p<0.05) and fewer apoptotic cells (p<0.05) than those from frozen semen.

**Conclusion:**

These findings suggest that 0°C refrigerated semen is superior to liquid nitrogen frozen semen in terms of sperm quality parameters and *in vitro* embryo development.

## INTRODUCTION

The value of Simmental cattle, as a globally widely promoted meat and dairy breed, is reflected in the multidimensional economic benefits and ecological adaptability [1]. As a core tool for animal genetic resource conservation, semen preservation technology is directly linked to breeding efficiency. According to statistics from the Food and Agriculture Organisation of the United Nations (FAO) in 2024, the global animal gene bank now holds over 120 million doses of frozen semen, with bovine semen accounting for 68%, porcine semen for 19%, and ovine semen for 13%. Since the discovery of glycerol as a cryoprotectant in the 1950s, numerous methods have been employed to enhance semen quality, including the use of various supplements, freezing techniques, and cryoprotectants [2,3]. Optimising semen preservation technology plays a crucial role in the genetic improvement and breeding of Simmental cattle. In livestock production, semen preservation is primarily divided into low-temperature preservation (4°C) and cryopreservation (−196°C). Cryopreserved semen (−196°C) is widely used due to its long-term storage capability; however, the decline in vitality after thawing remains the core issue limiting conception rates [4]. Studies have shown that the vitality of thawed frozen bovine semen is mostly concentrated between 45% and 55% [5]. In contrast, although low-temperature storage (4°C) can maintain short-term sperm activity, the acrosome integrity rate decreases by 30%–40% after 48 h, significantly affecting fertilisation efficiency [6]. In recent years, large quantities of frozen semen have been used in production. Although liquid nitrogen frozen semen has the advantage of long-term storage, the damage caused during the freezing process is irreversible [7]. Compared with cryopreservation, 0°C does not require the extreme process of −196°C [8]. 0°C serves as the critical temperature for freezing and refrigeration, enabling semen to remain in a state of ice-water mixture. Although this preservation method is less convenient than liquid nitrogen freezing in terms of preservation effectiveness, it has advantages over low-temperature preservation during transportation. *In vitro* fertilisation (IVF) as a core technology in modern animal breeding has transitioned from laboratory research to industrial-scale application in the livestock industry. Currently, the quantity and quality of *in vitro* embryos remain constraints on productivity development. Optimising the semen preservation technology system is essential to ensure the production of *in vitro* embryos.

Low-temperature semen storage at 0°C represents a critical yet understudied area in reproductive biotechnology, requiring a balance between metabolic suppression and cold-induced cellular stress. While traditional sperm storage typically uses temperatures between 4°C and 17°C to inhibit bacterial proliferation and maintain sperm viability [8], the feasibility of 0°C storage remains controversial due to increased cold shock risk. 0°C storage holds significant agricultural implications. In livestock breeding, it can bridge the gap between short-term refrigeration (≤24 h) and long-term cryopreservation, enabling antibiotic-free semen transport without the limitations of liquid nitrogen cryopreservation [9].There are currently insufficient systematic studies on the developmental potential of oocytes by means of semen preservation, and in particular relatively few studies have been carried out on 0°C refrigerated bovine semen, leading to even more sparse data on the resulting *in vitro* fertilised embryos [10]. We employed a self-formulated diluent for bovine semen refrigerated at 0°C. Sperm motility, progressive motility, acrosome integrity, and plasma membrane integrity were assessed between Simmental cattle liquid nitrogen-frozen semen and 0°C refrigerated semen, as well as the number of embryos generated from both methods. Providing data support for optimising germplasm conservation and promoting cost reduction and efficiency in the cattle breeding industry.

## MATERIALS AND METHODS

### Experimental procedure

First, we collected semen from six bulls. The collected semen was processed and then preserved in liquid nitrogen and at 0°C for 72 h, respectively. Subsequently, computer-assisted sperm analysis (CASA) was performed to evaluate sperm quality parameters, and the integrity of the acrosomal membrane was assessed. Concurrently, the levels of malondialdehyde (MDA), reactive oxygen species (ROS), adenosine triphosphate (ATP), and mitochondrial membrane potential (MMP) were quantified. Thereafter, ovaries were collected from a commercial abattoir, and subsequent IVF experiments were conducted using the aforementioned biological materials. The cleavage rate was observed at 48 h after IVF and the blastocyst rate was counted at day 8, then the embryos were observed by immunofluorescence to detect genes related to proliferation and apoptosis.

### Media and reagents

Tissue culture media (TCM)−199 medium, gentamycin, and phosphate-buffered saline (PBS) were purchased from Life Technologies Europe. All other chemicals not otherwise listed were obtained from Sigma-Aldrich. All media were filtered before use (0.22 μm; GE Healthcare-Whatman).

### Laboratory animals

Select six Simmental bulls (3–5 years old) in good physical condition with excellent reproductive performance. Collect semen from Simmental bulls once per week. Semen collection is performed using an artificial vagina method at 9 a.m. every Monday morning. Semen samples underwent visual and microscopic examinations to assess color, odor, and ejaculate volume, and these parameters were recorded. Microscopic analysis evaluated semen quality. Fresh semen that met the initial normal criteria was screened using a fully automated semen analyzer to select samples meeting the criteria of ≥75% motility, ≥6×10^8^/mL concentration, and ≤15% abnormal morphology.

### Semen collection and processing

In this experiment, semen was collected from six breeding bulls using the artificial vagina method at the Dingxin Breeding Bull Farm in Xinhe County, Xinjiang, China. Following collection, an initial motility assessment was performed through a subjective visual evaluation of sperm motility. The semen from each bull was subsequently divided into two aliquots: one portion was diluted with a cryopreservation extender (020996; Optidyl, Kasu Biotechnology). After dilution, the semen was loaded into straws (BOU023892; Clear Mini Straw, Kasu Biotechnology) and processed into frozen semen using a semen freezing machine (DT-LX0; Detou Temmoku). The cryopreservation procedure involved placing the tubed semen samples into a 4°C semen freezing machine for a two-hour equilibration period. Subsequently, liquid nitrogen vapor was employed for rapid freezing, reducing the temperature from 4°C to −120°C within 7 min, and maintaining it at −120°C for 10 min. The semen was then swiftly transferred into liquid nitrogen for cryogenic storage. The other portion was diluted with a laboratory-formulated refrigeration extender at 0°C and stored at this temperature 72 h (The 24 h refrigerated storage of semen fails to meet the practical requirements for *in vitro* embryo production; therefore, we have extended the storage period to 72 h.). We employed a self-formulated diluent for bovine semen refrigerated at 0°C (Patent No. ZL2007100181613, IPC Classification No. CN100581553C) [10]. The diluent formulation consisted of: Tris (hydroxymethyl) aminomethane (T6061T, Tris; Biotopped Biotechnology), 5.25 g; Citric acid (BZ54859; Biotopped Biotechnology), 2.02 g; Fructose (F6100D; Biotopped Biotechnology), 3.22 g; fresh egg yolk, 50 mL; Penicillin-Streptomycin Solution (Top0016S; Biotopped Biotechnology), total dose 600,000 IU; and sterile water, 200 mL, which were gently stirred for 30 min. The post-dilution sperm concentration was maintained at 40 million/mL in all samples. In this study, we placed semen samples in a container (foam box) filled with an ice-water mixture maintained at 0°C. This mixture can be stably maintained at 0°C under standard atmospheric pressure. We set the refrigerator temperature to 0°C, placed the ice-water mixture container inside, and conducted regular checks every 4 hours to ensure the temperature remained stable around 0°C throughout the entire 72 h storage period.

### Thawing of semen

Upon thawing frozen semen, it is removed from liquid nitrogen and rapidly placed in a 38°C water bath while being gently shaken for 30 seconds. Subsequently, the semen is collected into centrifuge tubes for further use. For semen that has been refrigerated at 0°C, the procedure involves taking it out, placing it in a 38°C water bath with gentle shaking for 30 seconds, and then collecting it into centrifuge tubes for application.

### Computer-assisted analysis

After 72 h of semen preservation, the sperm motility parameters were analyzed using the CASA system (ML-500JZ; Marang) in conjunction with an inverted microscope (TS100-F; Nikon). Prior to analysis, sperm samples underwent washing via centrifugation. An equal volume of PBS (P1020; Solarbio Science & Technology) was added to the semen in a centrifuge tube, gently mixed, and centrifuged at 300×g for 3 min. The supernatant was discarded, and this washing process was repeated twice. A 10 μL aliquot of semen was carefully injected into a 20 μm depth sperm counting plate (ML-CASA20-4; Mailang), ensuring that no air bubbles formed. The slide was then placed on a microscope stage preheated to 37°C. The illumination intensity of the microscope was adjusted for optimal visualization on the monitor. Sperm trajectories were captured at 60 Hz (0.5 seconds, 30 frames) to measure sperm concentration, motile sperm ratio, and CASA parameters, including three velocity metrics: curvilinear velocity (VCL), straight-line velocity (VSL), and average path velocity (VAP). Observations were conducted from at least five randomly selected fields, with triplicate repeats for each sample.

### Sperm acrosome and plasma membrane integrity detection

The FITC-PNA/DAPI staining method (MP6327; MX4208; Maokang Biotechnology) was employed in this study. Initially, 20 μL of sperm from various groups was placed on a glass slide, air-dried, and fixed with a 2% paraformaldehyde solution (MM1515; Maokang Biotechnology) for 10 min, followed by air-drying. Subsequently, 2 mL of PBS was added to the slide and incubated for 10 min, being repeated twice to eliminate residual paraformaldehyde. Next, 30 μL of the FITC-PNA working solution was evenly applied to cover the sperm sample, incubated in darkness at 37°C for 30 min, and washed three times with PBS to remove excess dye. Following this, 30 μL of DAPI staining solution was uniformly applied to the slide, incubated in darkness at room temperature for 5–10 min, and immediately observed and imaged under a fluorescence microscope (Nikon Ti2-U), ensuring that light exposure was strictly avoided throughout the process. Sperm exhibiting complete bright green fluorescence in the anterior acrosome were classified as intact acrosome sperm, whereas those with incomplete or absent fluorescence were considered acrosome-damaged. Imaging was conducted in complete darkness. The acrosome integrity rate was calculated as follows: Acrosome integrity rate (%) = (number of intact acrosome sperm / total sperm count)×100%. For plasma membrane integrity assessment, the SYBR 14/PI staining method (MX4239; MX4205; Maokang Biotechnology) was utilized following the same protocol as the FITC-PNA/DAPI staining. Sperm exhibiting green fluorescence were identified as viable, while red fluorescence indicated a loss of membrane integrity. The membrane integrity rate was calculated as follows: Membrane integrity rate (%) = (number of membrane-intact sperm / total sperm count)×100%. Sperm were observed across at least five randomly selected fields, and each sample was analyzed in triplicate.

### Sperm malondialdehyde content test

The MDA content in the samples was determined using an MDA detection kit, and the operation process strictly followed the instructions in the kit manual. The MDA content was calculated by plotting a standard curve. Each sample underwent three independent tests, and the average value was taken as the final result. The kit was manufactured by Nanjing Jiancheng Bioengineering Institute. Based on our experimental data, the upper and lower limits of this assay are 14.83–30.08 nmol/mL, with an inter-assay CV of 4.11% and an intra-assay CV of 6.24%.

### Sperm reactive oxygen species content test

Take 20 μL of thawed semen sample, immediately use the base dilution solution preheated at 37°C to dilute 1:6, take 50 μL of diluted semen and mix it with 2 μL of DCFH-DA probe, and incubate for 30 min at 37°C, protected from light. After incubation, wash the precipitate with PBS and resuspend it in 400 μL of PBS, and then measure the fluorescence intensity at 488 nm excitation wave and 525 nm emission wave by multifunctional zymography. After incubation, the precipitate was washed with PBS and resuspended in 400 μL of PBS, and the fluorescence intensity at 488 nm and 525 nm was measured by multifunctional enzyme marker.

### Sperm adenosine triphosphate content test

The ATP content in the samples was determined using an ATP detection kit, and the operation process strictly followed the instructions in the kit manual. The ATP content was calculated by plotting a standard curve. Each sample underwent three independent tests, and the average value was taken as the final result. The kit was manufactured by Solabio. Based on our experimental data, the upper and lower limits of this assay are 14.5–22.3 μmol/mL, with an inter-assay CV of 7.21% and an intra-assay CV of 5.59%.

### Sperm mitochondrial membrane potential content test

The MMP content in the samples was determined using an MMP detection kit, and the operation process strictly followed the instructions in the kit manual. The MMP content was calculated by plotting a standard curve. Each sample underwent three independent tests, and the average value was taken as the final result. The kit was manufactured by Solabio. Based on our experimental data, the upper and lower limits of this assay are 0.58–1.22 ΔΨm, with an inter-assay CV of 11.21% and an intra-assay CV of 12.43%.

### *In vitro* embryo production

The ovaries taken from the abattoir were washed three times using saline and oocytes were collected using a 9-gauge needle (During aspiration, better control of suction force reduces damage to cumulus cells, thereby yielding a greater number of oocytes). Only COCs with uniform cytoplasm and surrounded by at least three or more compact layers of cumulus cells were selected. *In vitro* maturation was performed individually in petri dishes (60×15 mm; Thermo Fisher Scientific) containing 17 droplets of 20 μL of maturation medium (TCM-199 with 20 ng/mL epidermal growth factor and 50 μg/mL gentamycin) covered with 7.5 mL paraffin oil (SAGE oil for tissue culture, ART-4008–5 P; Cooper Surgical Company) for 22 h at 38.5°C in 5% CO_2_ in humidified air. For IVF, fertilization was done individually in droplets of 20 μL of IVF-Tyrode’s Albumin Lactate Pyruvate (IVF-TALP) medium covered with 7.5 mL paraffin oil. To do so, the sperm concentration was adjusted to 1×10^6^ spermatozoa/mL using IVF–TALP medium enriched with bovine serum albumin (BSA; Sigma A8806, 6 mg/mL) and heparin (25 μg/mL). Individual COCs were then transferred to each droplet and incubated for 21 h at 38.5°C in 5% CO_2_ in humidified air. After 21 h of fertilization, COCs from each dish were pooled and vortexed for 3 min. Cumulus-free presumed zygotes were washed 3 times in a synthetic oviductal fluid (SOF) medium and individually transferred to 20 μL SOF droplets supplemented with 0.4% BSA (Sigma A9647), insulin, transferrin, and selenium (5 μg/mL insulin, 5 μg/mL transferrin, and 5 ng/mL selenium), and covered with 7.5 mL paraffin oil. Afterward, presumed zygotes were incubated at 38.5°C for 8 days in 5% CO_2_, 5% O_2_, and 90% N_2_. The control consisted of semen from two different preservation methods, which were added to the microdroplet during *in vitro* fertilization. *In vitro* culture was done in groups of 25 zygotes in droplets of 50 μL of SOF medium covered with 900 μL of paraffin oil. In all cases (group and individual culture systems), cleavage rate was determined at 48 h post insemination as the percentage of cleaved embryos out of presumed zygotes. Blastocyst rates were assessed on days 8 post insemination as the percentage of blastocysts out of presumed zygotes.

### Detection of proliferation and apoptosis in embryos

Both proliferation and apoptosis assays were conducted using grade 1 Blastocyst. Proliferation rates in the blastocyst were assessed via the BrdU assay. The collected embryos were washed three times with PBS buffer and fixed in a 4% paraformaldehyde solution for 30 min. After being transferred to PBS three times, the embryos were treated with 0.5% acid Tyrode’s solution for 5 min. Following three additional washes with PBS, the embryos were transferred to 0.5% Triton X-100 for 30 min and washed thrice with PBS. Subsequently, the embryos were incubated overnight with BrdU antibody (1:100 dilution; B100-1μL, Sigma-Aldrich) at 4°C. After three washes with PBS, embryos were incubated with FITC-conjugated goat anti-rabbit IgG (1:1,000; ab6939; Abcam) at room temperature for 2 h. Following thorough PBS washes, embryos were counterstained with DAPI for 5 min at room temperature. After three additional PBS washes, individual embryos were selected and placed on glass slides with 2 μL of anti-fluorescence quenching mounting medium (P0131; Beyotime Biotechnology). Cell proliferation rates were analyzed using a confocal laser scanning microscope (FV1000; Olympus Fluoview system). The proliferation index for each embryo was calculated by dividing the number of BrdU-positive nuclei by the total number of nuclei in the embryo. To assess apoptosis levels in embryonic, embryos were washed three times in PBS buffer and fixed in 4% paraformaldehyde for 30 min. After three PBS washes, embryos were treated with 0.5% acid Tyrode’s solution for 5 min, followed by three PBS washes and a 30 min incubation in 0.5% Triton X-100. Permeabilized embryos were incubated overnight with TUNEL solution in the dark at 4°C. Stained embryos were subsequently washed and incubated with DAPI for 5 min. After three PBS washes, individual embryos were selected and mounted on slides with 2 μL of anti-fluorescence quenching medium. Imaging was conducted using the confocal laser scanning microscope (FV1000; Olympus Fluoview system). The average percentage of TUNEL-positive nuclei per embryo was determined by dividing the number of positive nuclei by the total cell count (stained with DAPI). At least five randomly selected blastocysts were observed, and each sample was analyzed in triplicate.

### Embryo RNA extraction and detection of proliferation-and apoptosis-related genes

cDNA was reverse-transcribed and amplified from single blastocyst using the SMARTer Amplification Kit (634859; Takara Biomedical Technology). The relative mRNA transcript levels of all tested genes were analyzed via quantitative reverse transcription polymerase chain reaction (qRT-PCR). Proliferation-related genes, including Oct4 and Sox2, were detected, while apoptosis-related genes such as Caspase3 and Bax were also examined. The threshold cycle (Ct) values of all tested genes were normalized to the Ct value of GAPDH, with three replicates established for each sample. The relative expression levels of genes were calculated using the 2-ΔΔCt method. PCR amplification was performed under the following conditions: initial denaturation at 94°C for 5 minutes, followed by 40 cycles consisting of 30 seconds at 94°C, 30 seconds at 59°C, and 30 seconds at 72°C. GAPDH served as the reference gene in qRT-PCR, with the average expression level of GAPDH representing the expression level of the reference gene. Five independent experiments were conducted for gene expression analysis, each repeated 3 times. The primers used for qRT-PCR are listed in Table 1.

### Statistical analysis

Statistical graphs were generated using GraphPad Prism software ver. 8.0 (GraphPad Software). Data are presented as mean±standard deviation, based on a minimum of three independent experiments. Variables were compared between different groups using Student’s t-test (two-tailed). The fluorescence intensity of each image was quantified using ImageJ software. A p-value of less than 0.05 was considered statistically significant.

## RESULTS

### Computer-assisted analysis results

Through computer-assisted analysis, we found that the total motile sperm percentage of 0°C refrigerated sperm (86.60± 1.07) was significantly higher than that of liquid nitrogen-frozen sperm (76.52±1.09) (p<0.05). Similarly, the percentage of progressively motile sperm in 0°C refrigerated sperm (85.47± 1.21) was significantly greater than that of liquid nitrogen-frozen sperm (71.52±1.09) (p<0.05) (Figure 1A). Additionally, the values for VCL, VSL, and VAP of 0°C refrigerated sperm (61.87±5.09, 43.92±0.90, 40.93±0.90) were all significantly higher than those of liquid nitrogen-frozen sperm (51.31±3.10, 33.21±0.97, 32.29±0.96) (p<0.05) (Figure 1B).

### Detection of sperm acrosome and plasma membrane integrity

The sperm acrosome was stained with green fluorescence (Figure 2A). The integrity of the plasma membrane was assessed (Figure 2B). Statistical analysis revealed that the acrosome integrity rate of sperm refrigerated at 0°C (82.07±2.55) was significantly higher than that of sperm frozen in liquid nitrogen (75.80±1.78) (p<0.05). Similarly, the plasma membrane integrity rate of sperm refrigerated at 0°C (90.03±1.26) was significantly greater than that of sperm frozen in liquid nitrogen (83.11±2.96) (p<0.05) (Figure 2C).

#### Malondialdehyde and reactive oxygen species levels after cold storage

MDA is the final product of lipid peroxidation, and its concentration reflects the degree of lipid peroxidation. The production of MDA also exacerbates membrane damage [9]. As shown in Table 2, the MDA level changes with the change in storage method. The 0°C treatment group significantly reduced the MDA level of Simmental bull sperm (p<0.05). ROS mainly originate from the mitochondrial respiratory chain within cells and are produced by mitochondrial and enzyme reactions. As shown in Table 2, although refrigeration conditions still cause damage to sperm and produce a large amount of ROS, the ROS content after freezing and thawing is significantly higher than that after refrigeration and re-warmed (p<0.05).

### The effects of cold storage and freezing on adenosine triphosphate content and mitochondrial membrane potential in thawed sperm

As shown in Table 3, cold storage significantly increased the ATP content of sperm after freezing and thawing (p<0.05). The effect of refrigeration on the MMP of Simmental bull sperm after freezing and thawing is shown in Table 3. The results indicate that when semen is refrigerated at 0°C, the MMP of sperm is significantly increased (p<0.05).

### *In vitro* fertilization results

The results of IVF are shown in Figure 3. The cleavage rate at 48h was 77.15±2.99% in the 0°C group and 66.82±1.74% in the −196°C group, and the 48 h cleavage rate was significantly higher in the 0°C group than in the −196°C group (p<0.05). Observation of the day 8 blastocyst rate in both groups revealed that the blastocyst rate was significantly higher in the 0°C group (35.97±2.07%) than in the −196°C group (29.71± 2.46%) (p<0.05).

### Fluorescence staining detection of intracellular proliferation and apoptosis in embryos

Proliferating cells within the embryos were stained red using BrdU (Figure 4A), while apoptotic cells were stained green using TUNEL (Figure 4B). Statistical analysis indicated that the fluorescence intensity of proliferating cells in the 0°C group (26.99±1.57) was significantly higher than that in the −196°C group (22.11±1.51) (p<0.05). Conversely, the fluorescence intensity of apoptotic cells in the 0°C group (8.45±0.94) was significantly lower than that in the −196°C group (12.95± 1.37) (p<0.05) (Figure 4C).

### Detection of genes related to embryonic proliferation and apoptosis

The analysis of the expression levels of proliferation-related genes (Oct4, Sox2) and apoptosis-related genes (Caspase3, Bax) in embryonic cells (Figure 5) revealed the following findings: The expression levels of Oct4 and Sox2 in the 0°C group (1.38±0.10, 1.42±0.17) were significantly higher than those in the −196°C group (1.04±0.11, 1.00±0.05) (p<0.01). In contrast, the expression levels of Caspase3 and Bax in the 0°C group (0.79±0.13, 0.85±0.07) were significantly lower than those in the −196°C group (1.08±0.14, 1.14±0.15) (p<0.05).

## DISCUSSION

In this study, the percentage of motile spermatozoa and the percentage of forward-moving spermatozoa were significantly higher in the 0°C group compared to the liquid nitrogen freezing group (p<0.05), indicating that 0°C refrigeration is beneficial for spermatozoa during the preservation process of Simmental cattle spermatozoa. This finding is consistent with the results of previous studies [11]. During liquid nitrogen cryopreservation (−196°C), ice crystals formed from free water within sperm cells can puncture the plasma membrane and acrosome structure. In contrast, refrigeration at 0°C maintains a liquid environment, thereby preventing phase transition damage. This yields the same result across multiple species, including sperm cells from humans [12,13], salamanders [14], and buffalo [15]. Membrane integrity refers to the integrity of the outer membrane of sperm, which protects the internal structure of sperm from damage and helps maintain the structural integrity and function of sperm [16]. The acrosome is a special structure of spermatozoa that contains important enzymes and proteins involved in the binding and penetration of spermatozoa and ova [17]. Therefore, damage to the plasma membrane or acrosome during freezing may affect sperm motility, survival rate, and fertilisation capacity. This study also assessed the integrity of the plasma membrane and acrosome in Simmental cattle sperm following refrigeration, freezing, and thawing. With changes in storage conditions, the acrosome integrity rate (82.07% vs. 75.8%) and plasma membrane integrity (90.03% vs. 83.11%) significantly decreased (p<0.05), This is consistent with previous research [18], indicating that 0°C refrigeration has a positive impact on the fertilisation potential of Simmental cattle sperm.

MDA is one of the main products of lipid peroxidation. When cells are exposed to an oxidative stress environment, lipid molecules are attacked by free radicals, undergo peroxidation reactions, and generate MDA [19]. The sperm plasma membrane is rich in polyunsaturated fatty acids, making it susceptible to lipid peroxidation, which leads to loss of membrane integrity and reduces the ability of sperm to bind to oocytes [20]. ROS are normal metabolic products produced in living organisms and play an important role in cell signalling, immune regulation, and cell proliferation [21]. Therefore, maintaining appropriate ROS levels is crucial for maintaining sperm health and function. The results of this experiment indicate that storing Simmental bull semen at 0°C significantly reduces ROS levels (p<0.05), suggesting that 0°C storage can reduce oxidative damage to sperm. Sperm from different mammalian species differ in the main metabolic pathways that maintain sperm motility and survival [22]. Mitochondria play an important role in sperm cells, as they are believed to be the source of ATP that maintains cellular homeostasis and motility. At the same time, mitochondria are also the main site of ROS production in sperm cells [23]. ATP, as the primary energy source within cells, plays a crucial role in sperm, providing the energy required for motility, penetration of the egg, and other physiological processes. Bovine sperm can generate ATP through both glycolysis and mitochondrial oxidative phosphorylation pathways [24]. The MMP refers to the potential difference between the inner and outer membranes of mitochondria. The assessment of MMP is the most reliable parameter reflecting sperm mitochondrial function and an indicator of mitochondrial energy status. In this study, when Simmental bull semen was stored at 0°C, it significantly increased sperm ATP content, and this effect decreased with changes in storage methods. The percentage of motile sperm and the percentage of forward-moving sperm in 0°C-stored semen were higher than in the liquid nitrogen group, a phenomenon that may be attributed to higher ATP content. Additionally, Research has found that mitochondria maintain basal metabolism under low-temperature conditions, which facilitates gradual ATP consumption compared to complete metabolic arrest observed in liquid nitrogen cryopreservation [25,26]. 0°C-stored semen significantly increased MMP in thawed sperm.

Optimising semen storage methods can enhance embryo development potential. The synergistic effects of 0°C refrigeration technology and IVF protocols warrant attention. In IVF, the storage time of refrigerated semen aligns closely with the oocyte maturation window, avoiding post-thaw vitality decline (percentage of gradually active sperm post-thaw in the refrigerated group: 85.47±1.21% vs. liquid nitrogen group: 71.52±1.09%). In IVF, the cleavage rate and blastocyst rate were significantly improved in the 0°C refrigeration group (p<0.05), indicating that sperm storage methods directly influence the developmental potential of fertilised eggs in later stages. Sperm refrigeration and freezing methods affect the developmental capacity of embryos after fertilisation. Our fluorescence staining experiments (focused on proliferation and apoptosis) and marker gene detection assay solutions validated this conclusion.

Semen is utilised within a few days of collection and does not require liquid nitrogen freezing. Research has found that sperm selected for thermotaxis in ICSI exhibit a higher percentage of blastocyst development and better implantation rates compared to non-thermotactically selected sperm [27]. This suggests that the condition of sperm prior to fertilization contributes to embryo development. Sperm endurance recovery (SER)-treated sperm increased embryo development and pregnancy rates; however, instead of selecting the best sperm, SER appears to improve the overall sperm quality increasing their chances to produce high-quality embryos [28]. Therefore, sperm quality contributes to embryo development. Storage at 0°C preserves sperm quality while increasing the number and quality of embryos produced through IVF. As the large-scale breeding of beef and dairy cattle becomes increasingly intensive, the demand for *in vitro* embryo production continues to grow. The subject of this study is the Simmental beef cattle breed. Given the potential differences in semen quality between beef cattle and dairy cattle, future research should consider the long-term storage of beef cattle liquid semen. Although 0°C refrigeration demonstrates significant advantages, the liquid environment plays a crucial role in sperm storage and recovery [29], its long-term storage efficacy remains limited, Long-term storage (>7 days) requires further validation. In the future, adding different additives could enhance its long-term storage efficacy, avoid cryopreservation-induced damage caused by liquid nitrogen, and achieve long-term storage, thereby establishing a new semen storage method.

## CONCLUSION

This study demonstrates that sperm refrigerated at 0°C exhibits significantly superior motility, progressive motility, acrosome integrity, and plasma membrane integrity compared to sperm frozen in liquid nitrogen. In addition, the cleavage rate and blastocyst rate of 0°C cold storage were also significantly better than those of liquid nitrogen-frozen spermatozoa in IVF, thus verifying the feasibility of using 0°C cold storage for *in vitro* embryo production.

## Figures and Tables

**Figure 1:**
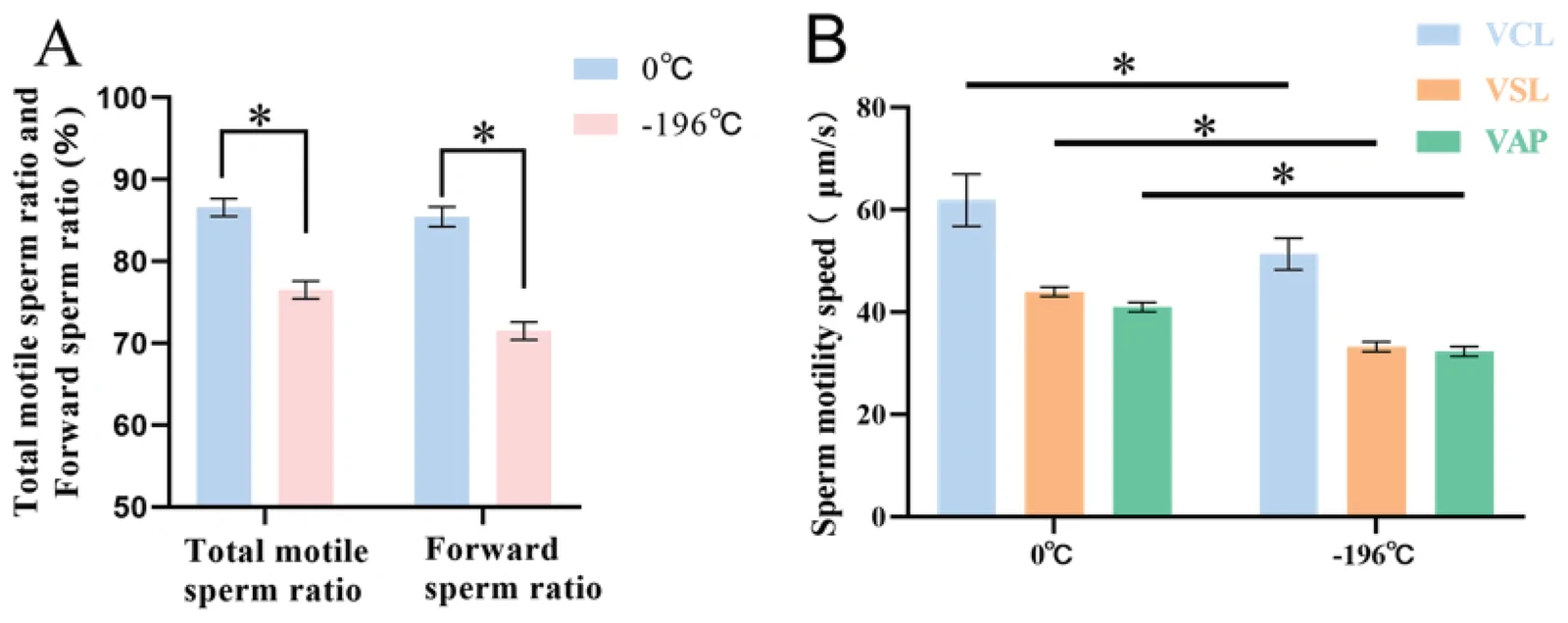
Results of computer-assisted sperm analysis. In the figure, "0°C" denotes the 0°C refrigerated sperm group, and "−196°C" represents the liquid nitrogen-frozen sperm group. * The asterisk indicates significant differences between the two groups (p<0.05) (n = 6). VCL, curvilinear velocity; VSL, straight-line velocity; VAP, average path velocity.

**Figure 2:**
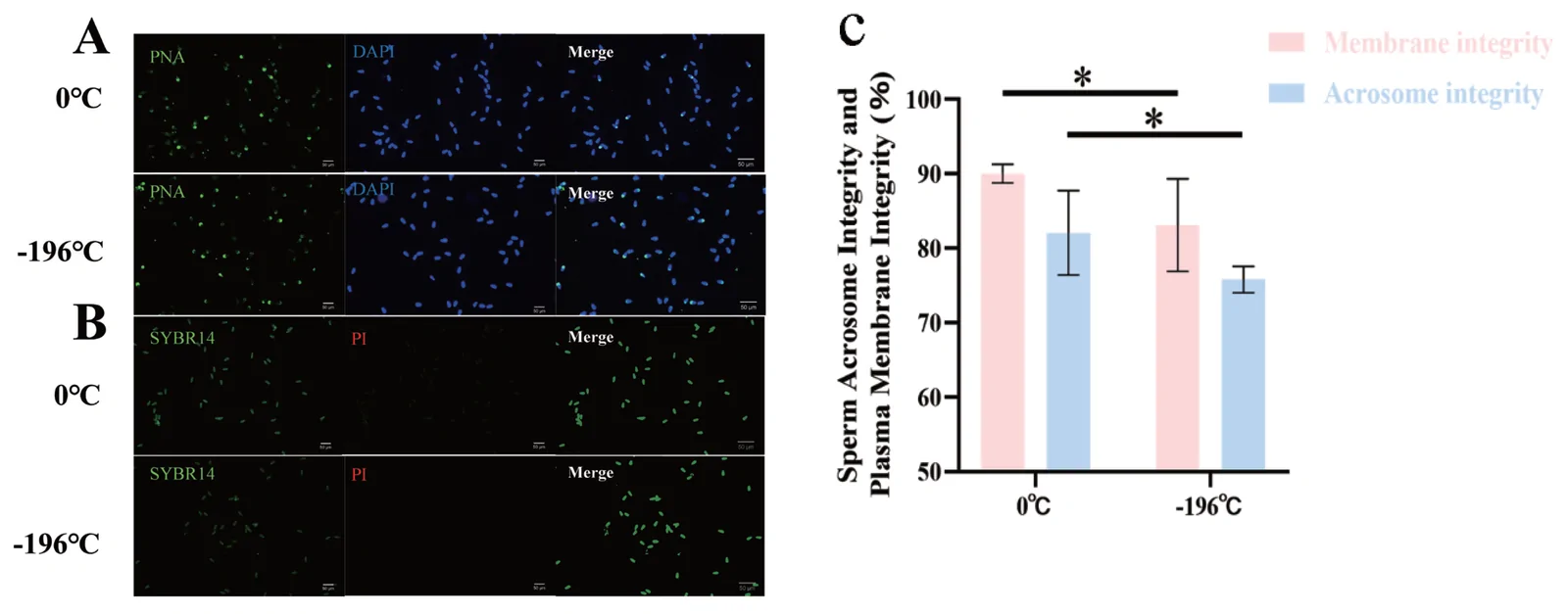
Results of sperm acrosome and plasma membrane integrity detection. (A) "0°C" denotes the 0°C refrigerated sperm group, and "−196°C" represents the liquid nitrogen-frozen sperm group (hereinafter the same). Green fluorescence indicates acrosome-intact sperm stained with FITC-PNA, while blue fluorescence represents DAPI staining. The scale bar is 50 μm. (B) Green fluorescence indicates viable sperm stained with SYBR 14, and red fluorescence represents PI staining (membrane-disrupted sperm). The scale bar is 50 μm. (C) * The asterisk indicates significant differences between the two groups (p<0.05) (n = 6).

**Figure 3:**
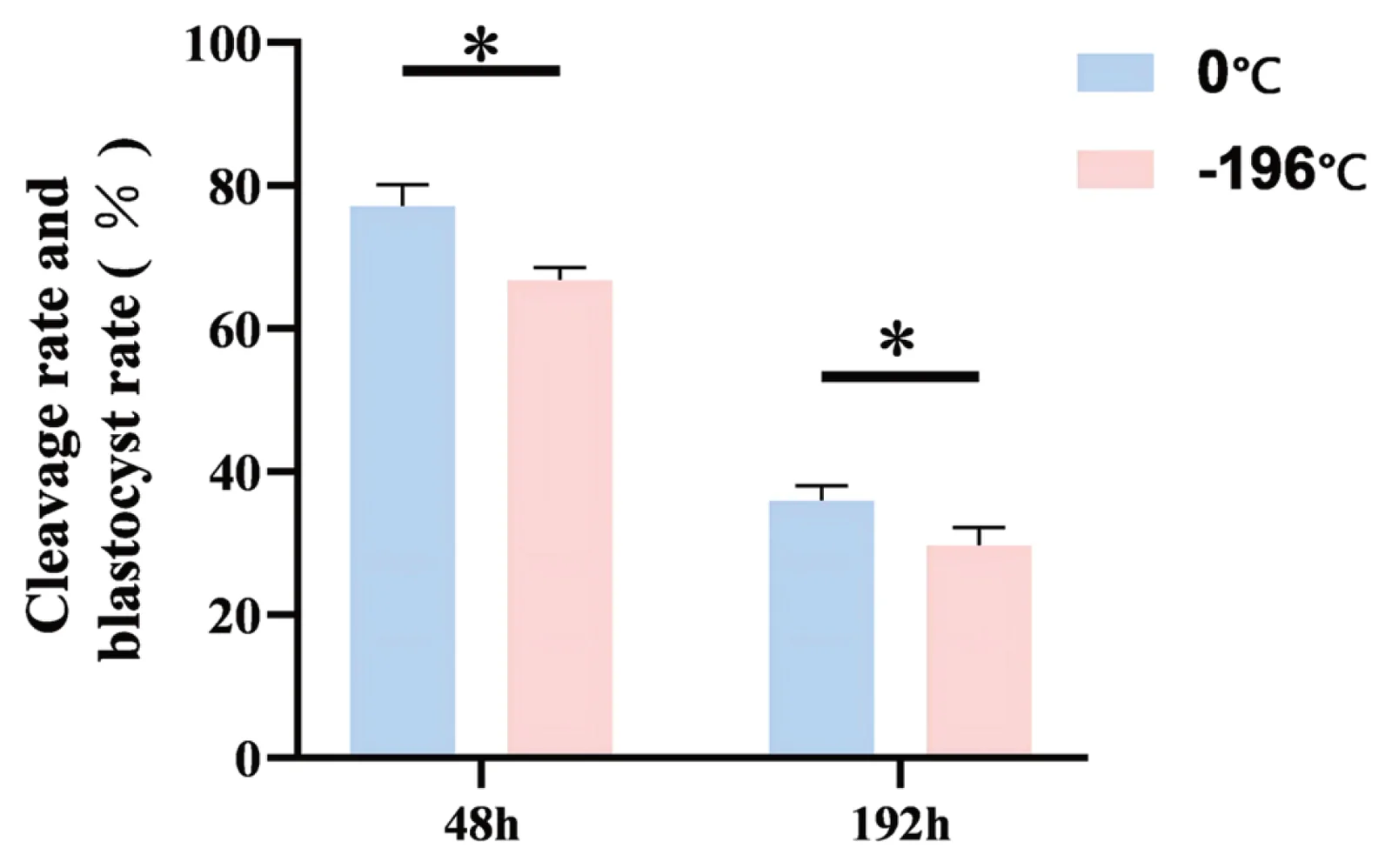
*In vitro* fertilization results. Each droplet contained 25 oocytes, with three droplets forming one group. Each group constituted one replicate, and a total of five replicates were performed. * The asterisk denotes a significant difference between the two groups (p<0.05).

**Figure 4:**
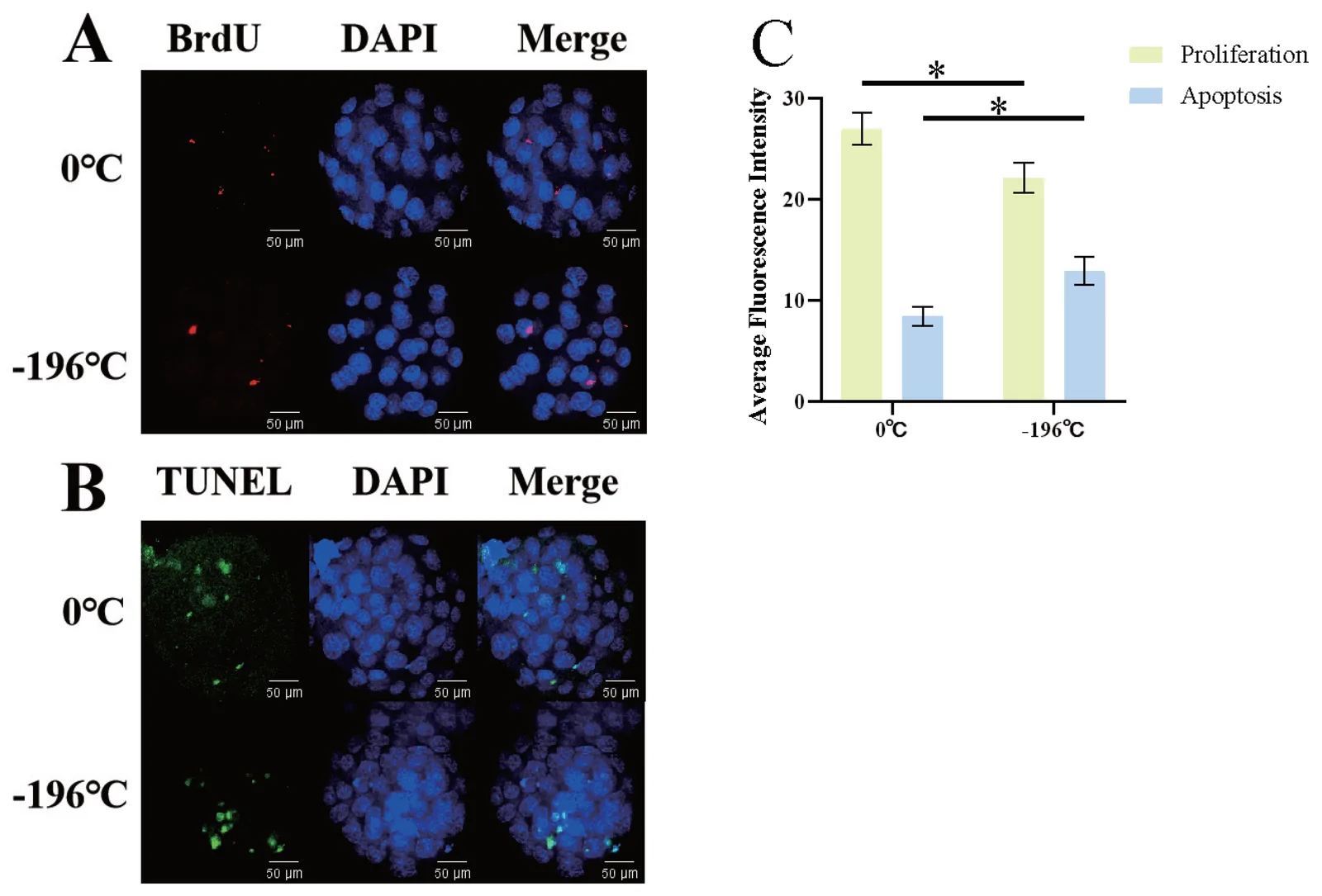
Fluorescence staining results of intracellular proliferation and apoptosis in embryos. (A) Red fluorescence indicates BrdU-stained proliferating cells within the embryo, and blue fluorescence represents DAPI staining; the scale bar is 50 μm. (B) Green fluorescence indicates TUNEL-stained apoptotic cells, and blue fluorescence represents DAPI staining; the scale bar is 50 μm. (C) * The asterisk denotes a significant difference between the two groups (p<0.05) (n = 6).

**Figure 5:**
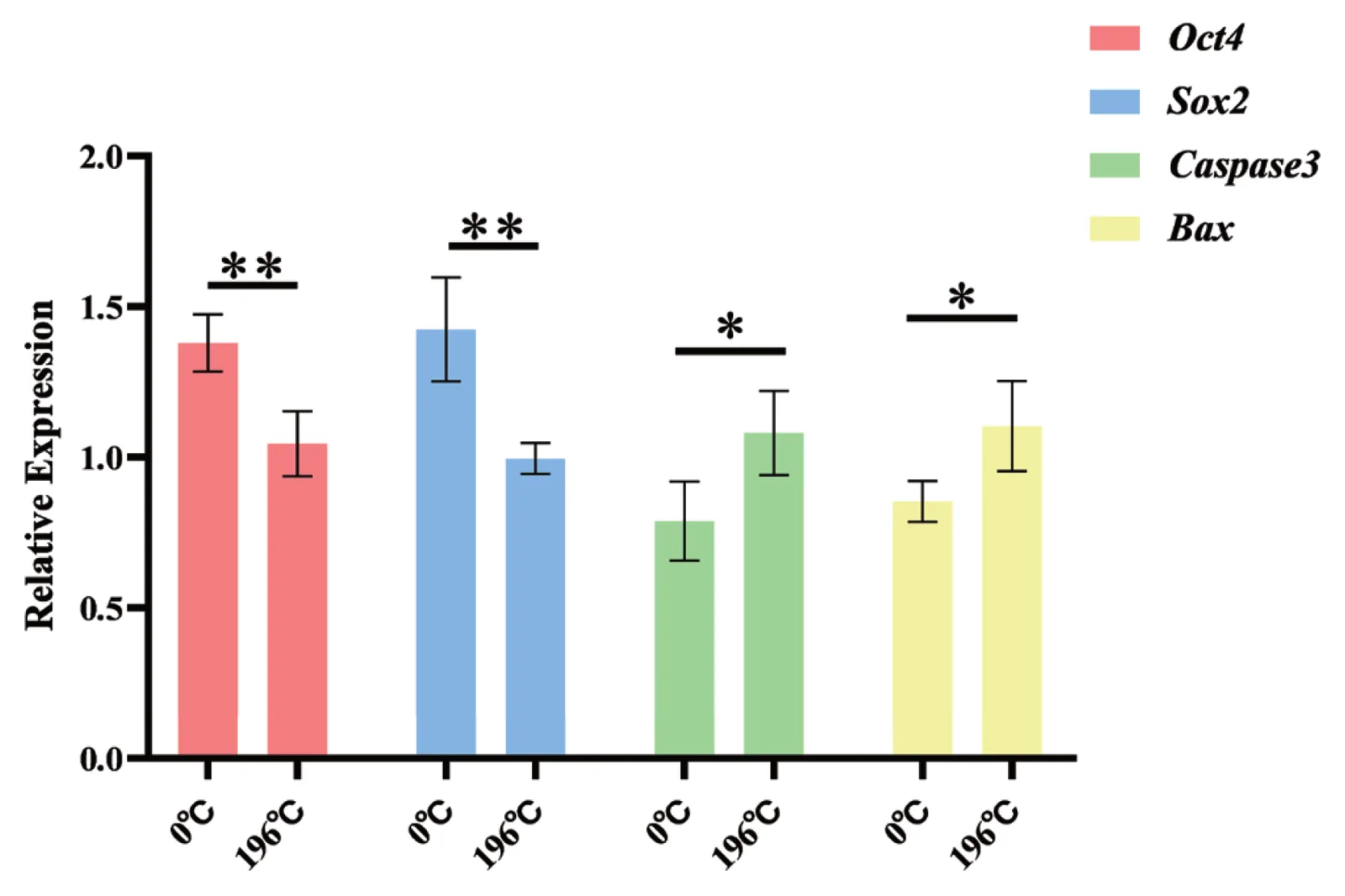
Test results of genes related to embryonic proliferation and apoptosis. * indicates a significant difference between the two groups at p<0.05, and ** at p<0.01.

**Table 1:** List of qRT-PCR primers

Gene name	Sequence	Annealing temperature (°C)	Product length (bp)
*Oct4*	F: GGGCAAACGATCAAGCAGTG	60	172
	R: CTCAGGGAATGGGACCGAAG		
*Sox2*	F: AGAGTGTTTGCAAAAGGGGGA	60	147
	R: CGCCGCCGATGATTGTTATT		
*Caspase3*	F: AGCGTCGTAGCTGAACGTAAA	59	247
	R: CTGCATCCACGTCTGTACCA		
*Bax*	F: ACAGGGGCCCTTTTGCTTCA	60	112
	R: TCTCGGGGAGAGTCTGTGTC		
*GAPDH*	F: CTGCCCGTTCGACAGATAGC	60	262
	R: TGATGACGAGCTTCCCGTTC		

qRT-PCR, quantitative reverse transcription polymerase chain reaction.

**Table 2:** ROS and MDA content of rewarmed sperm

Temp. (°C)	MDA (nmol/mL)	ROS (RFU)
0	16.37±1.11^a^	12,509.55±1,364.51^a^
−196	28.01±1.60^b^	34,193.45±1,525.90^b^

a,b The different letters in the same column indicate significant difference (p<0.05) (n = 6).

ROS, reactive oxygen species; MDA, malondialdehyde; RFU, relative fluorescence units.

**Table 3:** ATP content and MMP of sperm after rewarmed

Temp. (°C)	ATP (μmol/10^6^ sperm)	MMP (ΔΨm)
0	19.88±0.92^a^	1.06±0.11^a^
−196	16.37±1.07^b^	0.76±0.11^b^

a,b The different letters in the same column indicate significant difference (p<0.05) (n = 6).

ATP, adenosine triphosphate; MMP, mitochondrial membrane potential.

## Data Availability

Upon reasonable request, the datasets of this study can be available from the corresponding author.
